# Equally probable positive and negative Poisson's ratios in disordered planar systems

**DOI:** 10.1039/c8sm00717a

**Published:** 2018-07-27

**Authors:** Christophe M. Verstreken, Kevin J. Chalut, Raphael Blumenfeld

**Affiliations:** a Cavendish Laboratory , Department of Physics , University of Cambridge , Cambridge CB3 0HE , UK . Email: rbb11@cam.ac.uk; b Wellcome Trust/Medical Research Council Cambridge Stem Cell Research Institute , University of Cambridge , Tennis Court Road , Cambridge , CB2 1QR , UK; c Imperial College London , London SW7 2BP , UK

## Abstract

Auxetic materials, characterised by a negative Poisson's ratio, have properties that are different from most conventional materials. These are a result of the constraints on the kinematics of the material's basic structural components, and have important technological implications. Models of these materials have been studied extensively, but theoretical descriptions have remained largely limited to materials with an ordered microstructure. Here we investigate whether negative Poisson's ratios can arise spontaneously in disordered systems. To this end, we develop a quantitative description of the structure in systems of connected basic elements, which enables us to analyse the local and global responses to small external tensile forces. We find that the Poisson's ratios in these disordered systems are equally likely to be positive or negative on both the element and system scales. Separating the strain into translational, rotational and expansive components, we find that the translational strains of neighbouring basic structural elements are positively correlated, while their rotations are negatively correlated. There is no correlation in this type of system between the local auxeticity and local structural characteristics. Our results suggest that auxeticity is more common in disordered structures than the ubiquity of positive Poisson's ratios in macroscopic materials would suggest.

## Abstract 2

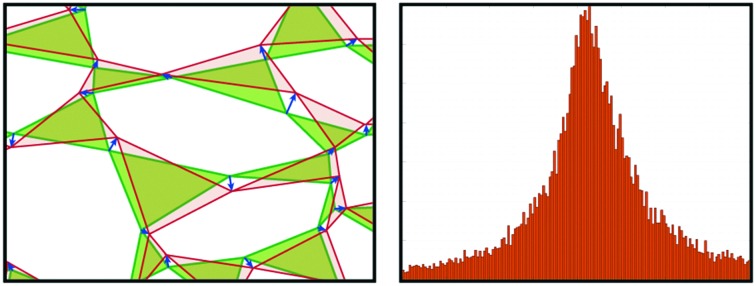
Disordered systems exhibit positive and negative Poisson's ratios with equal probability on the element and system scales.

## 1 Introduction

Materials with a negative Poisson's ratio (PR), called auxetics, have positively correlated horizontal and vertical strains, and thus expand (contract) in both directions when stretched (compressed).^1^ Various such materials can be found in nature, including polymers, foams, minerals, and even nuclei of stem cells.^2^,^3^ A range of auxetic structures has also been made artificially.^4^,^5^ Auxetic behaviour in disordered materials is not uncommon; for example, a crumpled ball of paper expands in all directions when stretched between two fingers. Yet, most investigations of the relation between internal structural characteristics and large scale auxeticity have focused on ordered systems.^6^–^13^ As such, few studies exist of auxeticity in disordered systems, including behaviour of three-dimensional folded sheets^14^,^15^ and perturbing slightly ordered structures by defects.^16^,^17^ Thus, auxeticity of disordered structures is still far from fully understood.

We address this problem by modelling auxeticity in isostatic structures that consist of minimally connected constituent units that can freely fold, expand and contract.^18^,^19^ Isostatic systems are statistically determinate—a particularly convenient property for our purposes, as we can relate the stress to the local microstructure by calculating the inter-element forces directly, without requiring elasticity theory. This feature also makes these structures ideal for modelling the jamming transition in systems in which the elements are macroscopic grains or colloids. We use arrangements of isostatic systems to analyse random disordered isostatic systems, and investigate the microscopic drivers of auxeticity on the local and global scales.

To be isostatic, a mechanically stable structure has only to satisfy a minimal connectivity criterion: that the mean number of force-carrying inter-element contacts per element is equal to a specific value *z*_*c*_.^20^ Generically, *z*_*c*_ = *d*(*d* + 1) and *d* + 1 for *d*-dimensional systems of frictionless and frictional non-spherical elements, respectively.^21^ Analysis of stress transmission in these materials explains the ubiquity of non-uniform stress states exhibited by particulate media.^22^–^25^ A first-principles continuum stress theory for two-dimensional isostatic granular media has been developed, based on a parameterisation of the inter-element forces into ‘loop forces’, where loops are the elementary voids, or cells, enclosed by individual elements.^26^,^27^

**Fig. 1:**
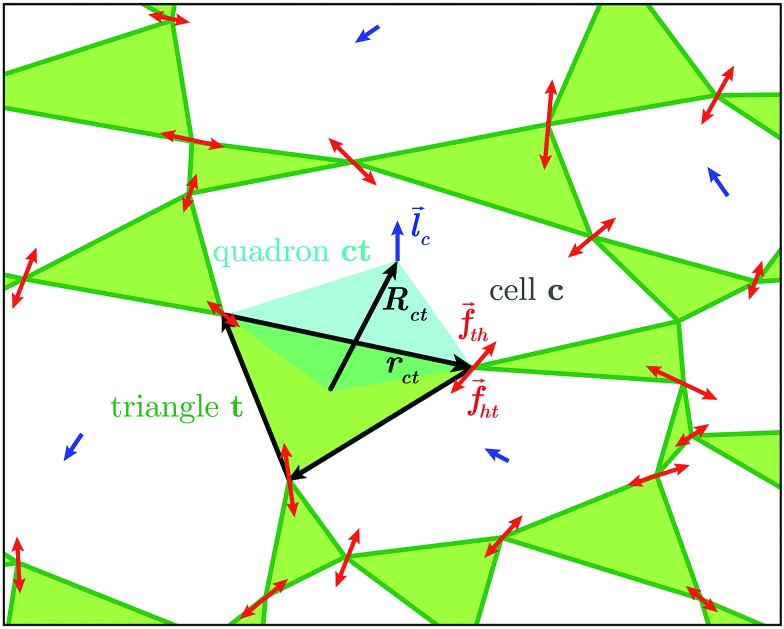
Close-up view of individual triangles in our system. The edges of triangle *t* are made into vectors, *r[combining right harpoon above]*_*ct*_, that circulate the triangle clockwise. These vectors make a disordered graph of randomly shaped triangles, connected to their nearest neighbours at the vertices. The vector *R[combining right harpoon above]*_*ct*_ extends between the centroid of triangle *t* and that of its neighbouring cell, *c*. The vectors *r[combining right harpoon above]*_*ct*_ and *R[combining right harpoon above]*_*ct*_ are the diagonals of a quadrilateral volume element, which is called quadron.^29^ Inter-triangle forces (red) are calculated as the difference between the loop forces *l[combining right harpoon above]*_*c*_ (blue) of neighbouring cells.

Since *z*_*c*_ = 3 in two dimensions, we choose to focus on planar systems of triangles, connected to nearest neighbours at the vertices by conceptual frictionless hinges, as illustrated in Fig. 1. For later use, we assign a direction to the edges of each triangle, *t*, making it a vector, *r[combining right harpoon above]*_*ct*_, with *c* being the cell that the edge borders. Thus defined, these vectors circulate clockwise around the triangles.^26^ For each *r[combining right harpoon above]*_*ct*_, we define a dual vector *R[combining right harpoon above]*_*ct*_, extending from the centroid of triangle *t* to the centroid of cell *c*. These two vectors form the diagonals of a quadrilateral, known as a quadron.^28^ The quadrons are fundamental volume elements that tessellate the system space perfectly. Moreover, the structure of each quadron can be quantified unambiguously by a local structural tensor, *r[combining right harpoon above]*_*ct*_⊗*R[combining right harpoon above]*_*ct*_, allowing us to quantify the local disordered structure anywhere in the system.^26^,^28^,^29^ This is convenient for relating local structural characteristics to local PR.

In mechanical equilibrium, the forces of triangles on their neighbours (inter-triangle forces or ITFs), can be parametrised using loop forces, with one loop force *l[combining right harpoon above]* per cell enclosed by triangles. As such, the force that triangle *t* exerts on its neighbour *t*′ is the difference between the loop forces of the two cells, *c* and *c*′, straddling the common joint of these triangles^26^ (see Fig. 1):

1 *f[combining right harpoon above]*_*tt*′_ = *l[combining right harpoon above]*_*c*′_ – *l[combining right harpoon above]*_*c*_

By construction the loop forces automatically satisfy force balance on each triangle, leaving only the torque balance conditions. This cuts by two thirds the number of equations to solve, considerably reducing the computational effort.

By calculating the fabric tensor, the quantitative description of the local structure of our isostatic systems can be coarse-grained to the overall system.^26^,^27^,^30^ Furthermore, the fabric tensor is related to local rotational strains,^31^ and can be used to identify and isolate rotational strains in auxetic materials.^18^,^19^ In this paper, we extend this latter development and apply it to random systems.

## 2 Theory

We first determine the number of equations required to calculate the inter-triangular forces in a disordered planar system consisting of *N* (≫1) triangles. The contours of the triangles form a graph of *N*_v_ connected vertices, *N*_e_ edges, and *N*_f_ faces. Identifying the *N*_b_ boundary triangles in this structure, it is convenient for the purpose of our analysis to enclose this graph within a frame to which the boundary triangles are connected by one of their vertices. For such a graph, Euler's topological relation for two-dimensional graphs in the plane is,^32^

2 *N*_v_ – *N*_e_ + *N*_f_ = 1,

where *N*_v_, *N*_e_ and *N*_f_ are the numbers of the graph's vertices, edges and faces, respectively.

In our graph, the vertices are the analogues of the contact points in granular assemblies. The edges are the triangle edges and the additional *N*_b_ edges formed by the frame. The faces consist of the triangle faces and the internal elementary voids (called cells or loops here), which include the additional *N*_b_ voids formed by the frame. Putting these numbers together, we have: *N*_f_ = *N*_c_ + *N* + *N*_b_ faces, (the *N*_c_ internal cells, the *N* loops around each triangle and the *N*_b_ boundary cells), *N*_e_ = 3*N* + *N*_b_, and *N*_v_ = (3*N* + *N*_b_)/2. Substituting these into Euler's relation (2), we have3
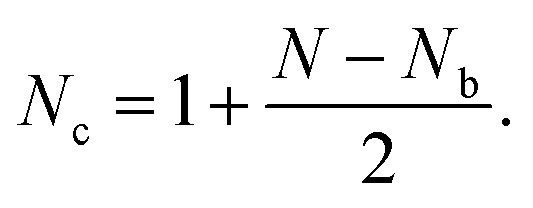
Since one loop force is associated with each cell, there are *N*_c_ + *N*_b_ loop forces altogether, which yield, using (3), 2 + *N* + *N*_b_ unknowns. Furthermore, each ITF is defined as the difference between two loop forces, meaning we can fix one of the loop forces at will.^26^ This reflects the nature of the loop forces field as a potential field, of which the local ITF is the gradient.^26^ Having exactly *N* torque balance conditions to determine the loop forces, we therefore need to fix *N*_b_/2 boundary forces on the vertices connected to the bounding frame. This leaves an exact set of equations to solve for the loop forces. We can then solve for the internal *N*/2 loop forces, from which we can determine the ITFs.

Specifically, we can define the following *N* × *N* matrix **A**, loop forces vector **X**, and boundary forces vector **B**.^33^ As before, the first *N* rows correspond to the torque balance on all *N* triangles, the next *N*_b_ rows correspond to the boundary forces on *N*_b_/2 vertices, and the final 2 rows normalise the 2 force components of the first loop force:4
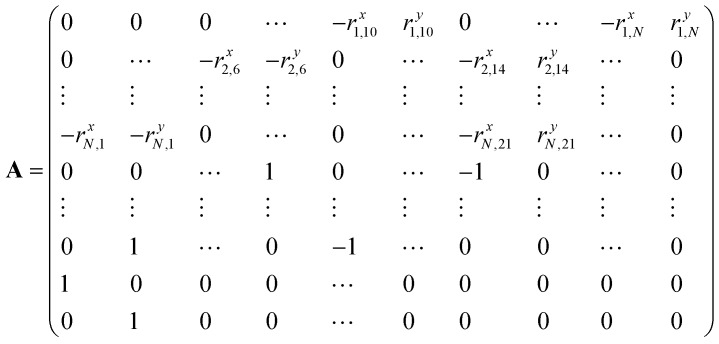

5
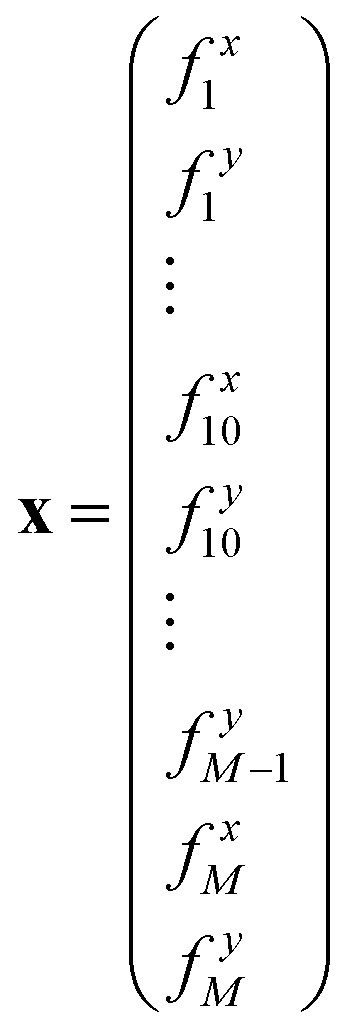
and6
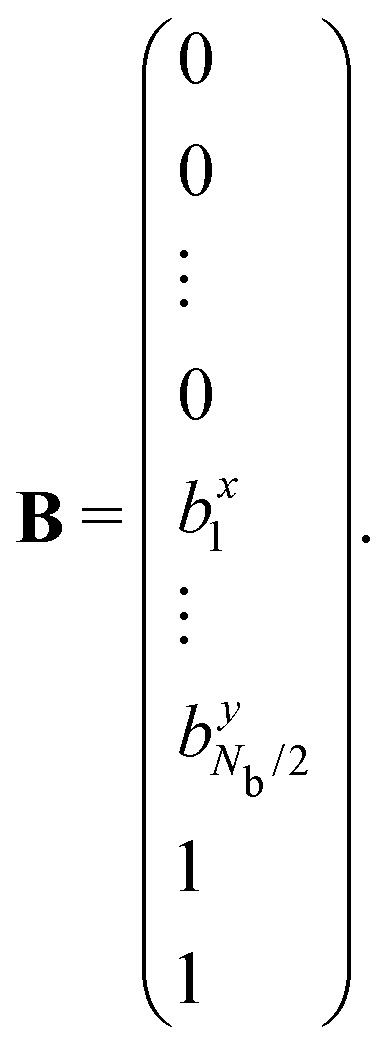
we have

7 **Ax** = **B**

We can solve for the loop forces by Gaussian elimination.

## 3 Model and methods

**Fig. 2:**
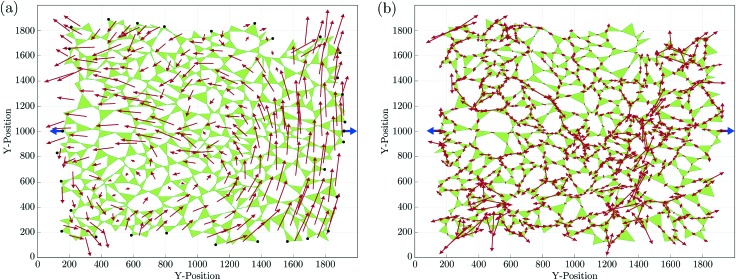
The force field response to two antipodal tensile forces, applied at the centre of opposite boundaries (in blue, not to scale). Shown in red are: (a) the loop forces, *l[combining right harpoon above]*_*c*_; (b) the corresponding ITFs.

We generated numerically 15 000 disordered planar assemblies of triangles as model systems. First, we distributed 200 points randomly within a rectangle of 2000 × 2000 in arbitrary units. We then generated a Voronoi tessellation around the points of the system, obtaining a planar cellular structure, with exactly three edges emanating from each node. Connecting the midpoints of these edges around each node produces a disordered assembly containing about 300 triangles in each system. With exactly 3 = *d* + 1 contacts per triangle, we made this system isostatic by imposing one force on every other boundary triangle vertex. We then used (7) to calculate the loop forces (see, *e.g.*Fig. 2a) and, from those, the ITFs by (1). Typically, the solutions contain regions in which the loop forces have smoothly-changing directional orientation, separated by sharp gradients, as illustrated on the right side of Fig. 2a. The sharp gradients correspond to localised force chains.^26^,^27^,^34^,^35^ These force chains can be observed experimentally in granular systems.^36^–^38^

Starting from the initial stress state, we calculated the mean force amplitude at a vertex for each system, *f[combining macron]*, and applied two equal and opposite small tensile forces, of amplitude about δ*f* = 1 × 10^–3^*f[combining macron]*, antipodally on the left and right boundaries, along the *x*-axis near the middle of the system (shown as blue forces in Fig. 2). We then calculated the changes in the loop forces and the ITFs.

**Fig. 3:**
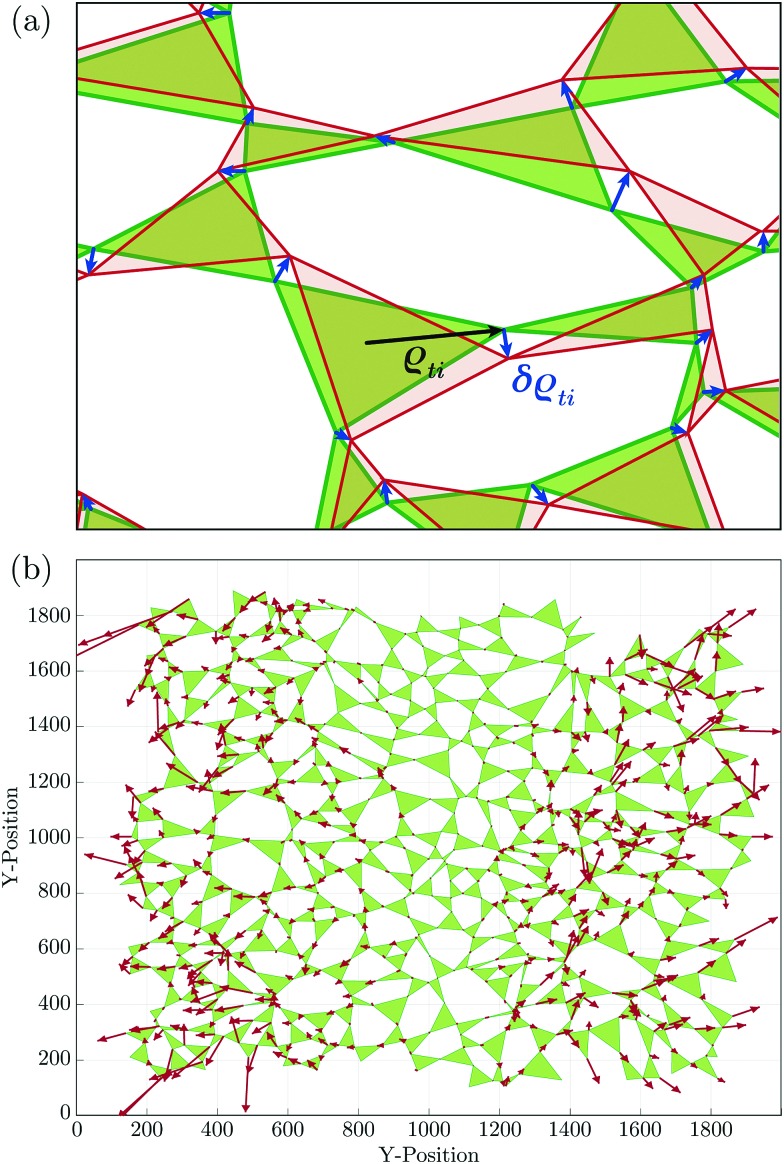
(a) Vector *ρ⃑*_*ti*_ points from the centroid of triangle *t* to its vertex *i*, and this vertex is displaced by δ*ρ⃑*_*ti*_. (b) Displacement of vertices in response to 100 small increments of the boundary forces.

The changes in the ITFs give rise to vertex displacements, resulting in triangle rotations, translations and expansions (or contractions), such that the triangle edges remain straight. It should be noted that the static determinacy constrains all the degrees of freedom and, therefore, the vertex displacements are only the result of the triangles’ elastic compliance. In the absence of inertial effects, each vertex's displacement is proportional to the force on it, and we expect vertices to displace towards the external tensile force it is closest to. This divides the system naturally into two halves with an imaginary half-line at *x* = 0. The vertex's displacement is then in the direction of the force pulling toward the closest boundary, scaled by a proportionality factor *ζ* that is 0 on the half-line and 1 on the corresponding boundary. A typical example of a displacement field is shown in Fig. 3a.

The displacements data were then recorded for later analysis, increased the magnitudes of the boundary tensile forces, calculate the new ITFs, and repeated the process. The accumulated displacements resulting from 100 successive repetitions of the process are shown in Fig. 3b.

In the following, we refer to the gradient of the displacement as strain, for short, although this term is normally reserved for its symmetric part. The strain can be defined for individual triangles and can be separated into a translational, an expansive and a rotational component:

8 *ε*_*t*_ = *ε*trans*t* + *ε*exp*t* + *ε*rot*t*.

In our system of non-rigid triangles, all these components are relevant,^19^ while in systems consisting of rigid triangles, only the translational and rotational components contribute to the total strain.^26^

The translational strain captures the displacements of the triangles’ centres of mass and it is defined as9
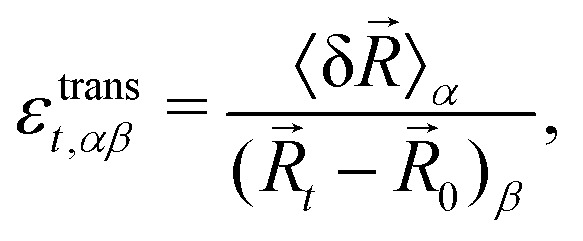
where *R[combining right harpoon above]*_*t*_ is the vector from the origin to the centroid of triangle *t*, *R[combining right harpoon above]*_0_ is the position of the entire system's centre of mass, and δ*R[combining right harpoon above]* is the mean displacement of the vertices of *t*. The expansive component of the strain depends on the outward or inward displacements of the vertices of the triangle, projected on the vector *ρ*_*ti*_10
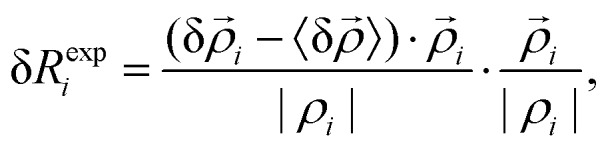
where *i* = 1, 2, 3 are the three vertices of the triangle. The expansive strain is then11
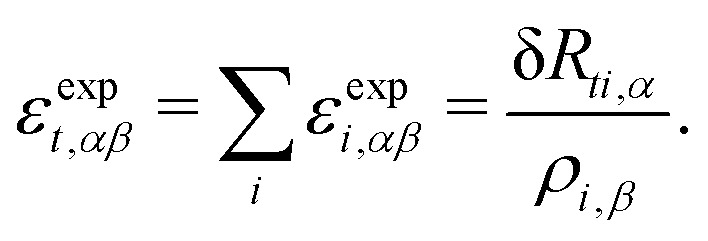



Each triangle hosts exactly three quadrons, as shown in Fig. 1, each described by a structure tensor **C**_*ct*_ = *r[combining right harpoon above]*_*ct*_⊗*R[combining right harpoon above]*_*ct*_.^28^ The triangle's structure tensor is the sum of the structure tensors of its three quadrons:12
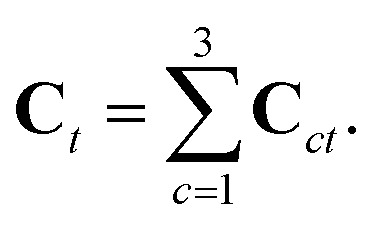
The antisymmetric part of this tensor gives the (generically, non-convex) area associated with the triangle's three quadrons, *A*_*t*_,13
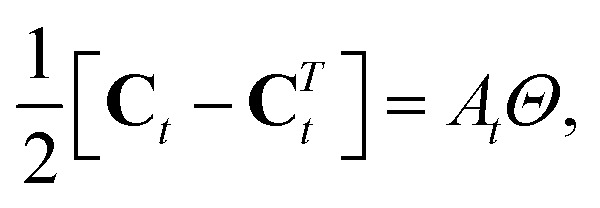
where **C***Tt* is the transpose of **C**_*t*_ and *Θ* is the two-dimensional π/2 rotation matrix (the Levi-Civita operator). The symmetric part of **C**_*t*_,14
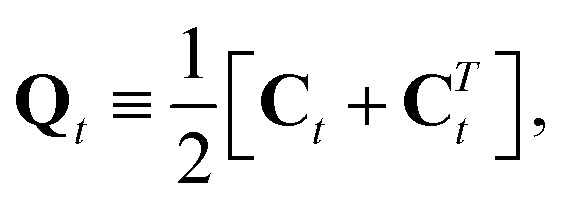
describes the local rotation of the triangle relative to the global mean.^26^,^27^ It has also been shown^31^ that the rotational strain satisfies15
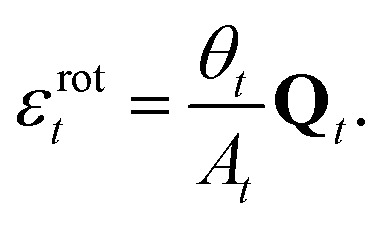
In terms of the triangle variables, the rotational component of the displacement is the displacement perpendicular to the direction of the expansion16
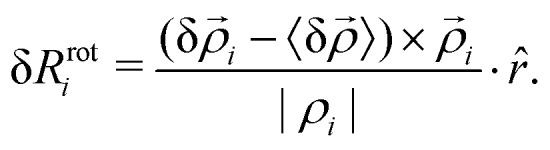
Therefore, the rotation angle of vertex *i* is17
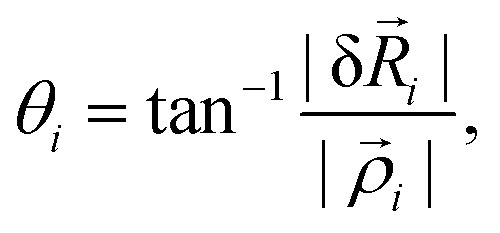
and the rotational strain of the entire triangle is18
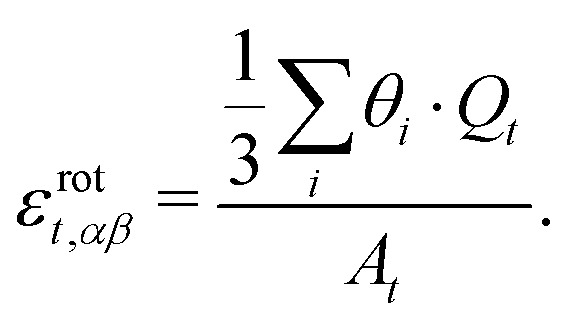



## 4 Results

Defining the PR of triangle *t* as

19 *ν*_*t*_ = *ε*_*t*,*yy*_/*ε*_*t*,*xx*_,

provides a local definition of the PR and makes it possible to study the spatial distribution of this property across the system.

**Fig. 4:**
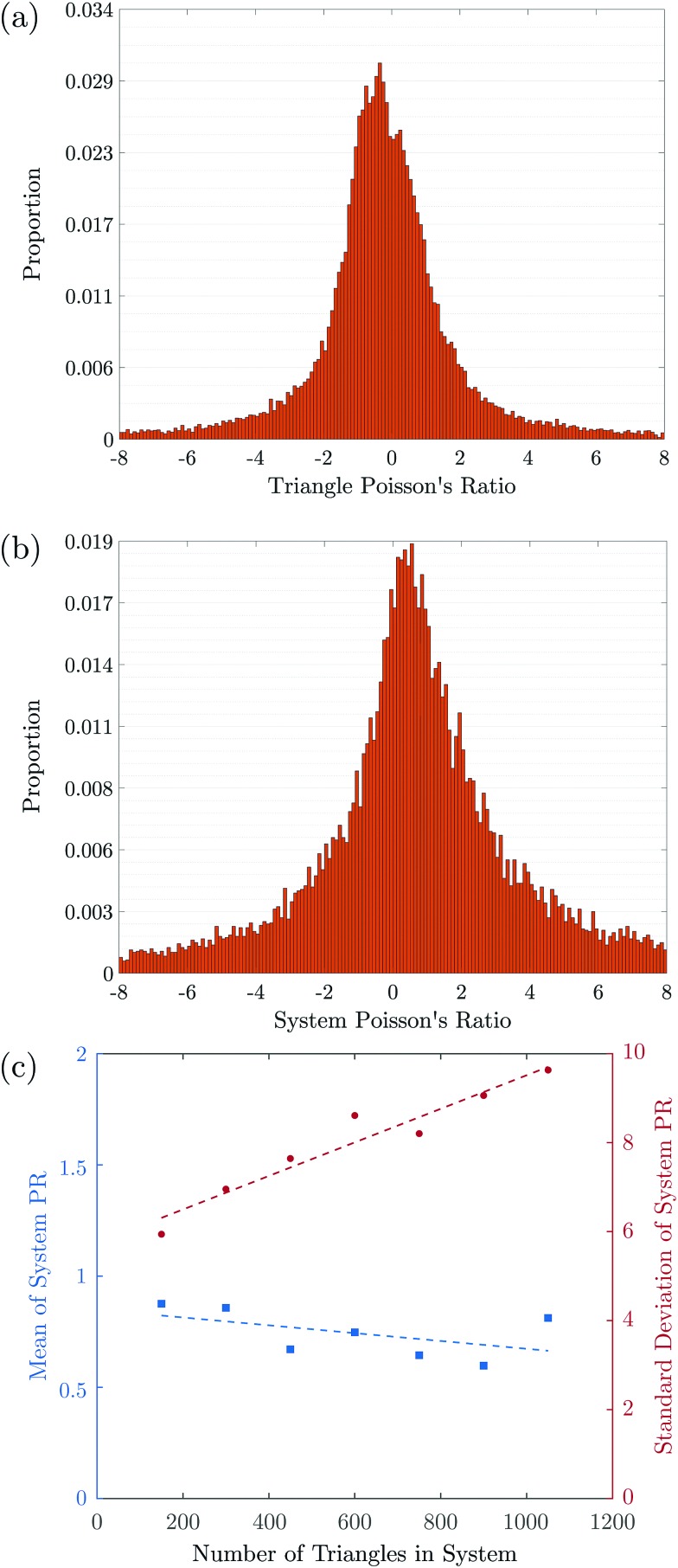
(a) Histogram of the PRs of individual triangles, *ν*_*t*_, calculated from over 40 000 triangles from 100 simulated systems. (b) Histogram of the PR of global systems, calculated from over 15 000 systems of 300 triangles each. (c) Mean (blue; left axis) and standard deviation (red; right axis) of system of 150 to 1050 triangles each. The mean is fitted well by PR = –1.8 × 10^–4^*N* + 0.8 and the standard deviation by *σ̄*_PR_ = 3.8 × 10^–3^*N* + 5.7, with *N* the number of triangles.

In Fig. 4a, we plot a histogram of the local PRs for data collected from over 40 000 triangles from 100 different system realisations. It is broadly symmetric, with the mean and standard deviation being –0.2 and 1.8, respectively, demonstrating that there is hardly any sign preference for the local PRs.

We also computed the global PR of each system, defined as20
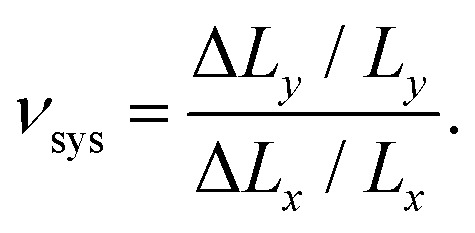
Here, *L*_*α*_ is the system extension in the *α* direction, defined as the difference between the mean positions of the vertices at opposite boundaries, and Δ*L*_*α*_ is the mean change in that distance. In Fig. 4b we show the histogram of *ν*_sys_ across 15 000 systems of 300 triangles each. This histogram is also symmetric, with its mean and standard deviation 0.9 and 7.0, respectively. Interestingly, this histogram's standard deviation is much larger than for the individual triangles, which is a result of its tails falling slower than exponential, although faster than algebraically.

To examine the dependence of these results on system size, we repeated the simulations for systems of sizes ranging from *N* = 150 to *N* = 1050 triangles each. We find that the distributions’ standard deviations are an order of magnitude larger than their means and are broadly symmetric around 0, as can be observed in Fig. 4. Zooming in on the means and standard deviations of the global PR distributions over the 7-fold size variation, we observe that the mean decreases slightly with *N*, from 0.82 to 0.66. This decrease is not statistically significant and is also consistent with no change within 95% confidence. In contrast, the standard deviation increases significantly with size by about 53%, from 6.31 to 9.70, indicating, with 95% confidence, that the width of the PR value distribution increases proportionally to *N*.

To gain insight into the relationship between the Poisson's ratio and the local structure, we investigated the local correlations between structural, elastic and strain properties, with the structural characteristics quantified using the above quadron description. To this end, we first express the stress on triangle *t* in terms of the loop forces of the cells surrounding it.^26^ The increase in the boundary forces gives rise to the following change in the stress21
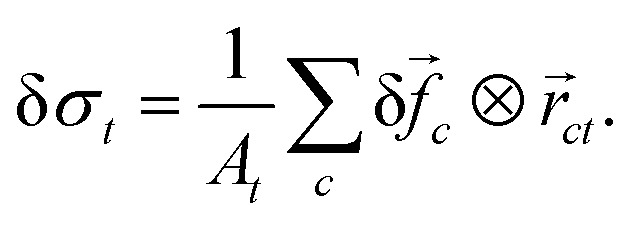
Given a triangle's stress and strain, we also calculated its compliance matrix, **S**_*t*_, using *σ*_*t*_ = **S**_*t*_*ε*_*t*_.

**Fig. 5:**
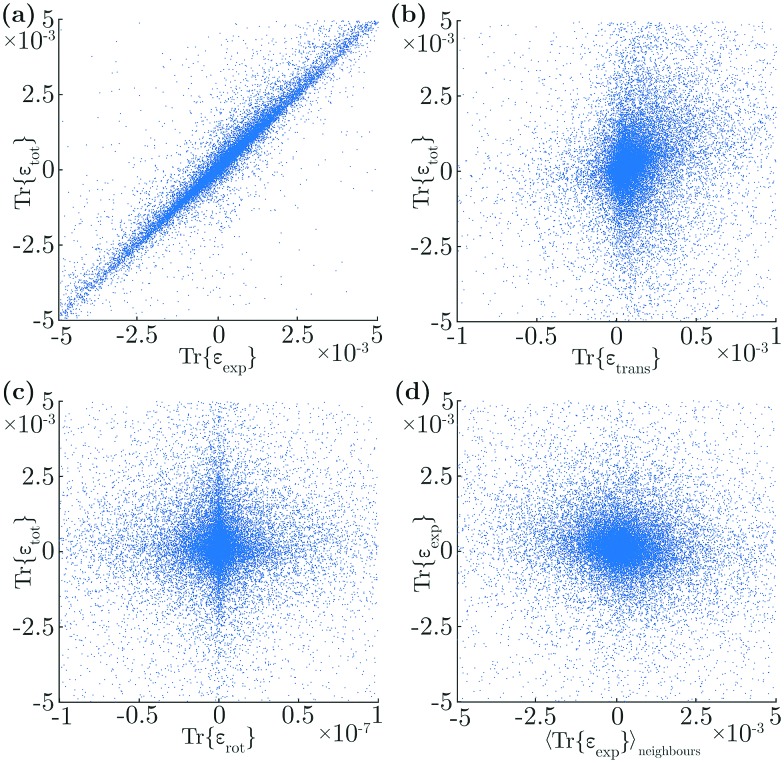
The relative contribution of strain components to the total strain of individual triangles. We show the correlation between the traces of a triangle's: (a) expansive and total strain; (b) translational and total strain; (c) rotational and total strain. In (d) we show correlation between the trace of a triangle's expansive strain and the average of the trace of the expansive strain components of the triangle's neighbours.

In particular, we correlated the traces of the different strain components of individual triangles with the trace of that triangle's total strain, for 100 systems and 3 × 10^4^ triangles. Irrelevant outliers, within the 2% highest and lowest strains, were disregarded. We find that the expansive component is the dominant contributor to the symmetric part of the strain, with a correlation of *γ* = 0.93 between Tr{*ε*exp*t*} and Tr{*ε*tot*t*} (Fig. 5a). The second largest contributor is the translational strain, with a correlation of *γ* = 0.24 between Tr{*ε*trans*t*} and Tr{*ε*tot*t*} (Fig. 5b). In contrast, there is hardly any correlation between the rotational component and the total strain, *γ* = 0.03 (Fig. 5c). As expected, the importance of expansion in the overall strain of individual triangles causes a negative correlation of *γ* = –0.10 between the expansive strains of neighbouring triangles (Fig. 5d). The translational strains of neighbours are positively correlated, with *γ* = 0.67, which is induced by our method of determining vortex displacements. Interestingly, while the rotational strain does not correlate well between nearest neighbours, with *γ* = –0.06, the rotation angle of neighbours is anti-correlated, with *γ* = –0.35, which is an aspect of the ‘anti-ferromagnetic’-like rotational nature of such systems.^11^,^31^,^39^–^41^

We also investigated the correlations between the one-triangle PR and local structural characteristics, including the triangle: size, shape, quadron sizes, displacement, and any of the strain contributors. We found no significant correlation between any of these quantities.

## 5 Conclusions and discussion

We studied the poorly understood issue of auxetic behaviour in disordered structures, modelled here by planar statically determinate, or isostatic, systems of triangular elements. Specifically, we addressed the question of whether a negative Poisson ratio (PR) can emerge spontaneously without design or whether a positive PR is more common, as its ubiquity in macroscopic materials might suggest.

We simulated 15 000 disordered planar systems, each of about 300 randomly-shaped connected compliant triangles, stretched them incrementally, and computed the PR for each triangle and for the entire assembly. Our main finding is that positive and negative PRs are almost equally likely to occur in such disordered systems. This conclusion holds on both the triangle scale (Fig. 4a) and the entire system (Fig. 4b). This suggests that large-scale systems with this layout would be almost PR-neutral, displaying a behaviour that is neither conventionally elastic nor fully auxetic. This result may seem counter-intuitive in view of the prevalence of positive PRs in macroscopic structures. However, it reinforces that these are not conventional elastic systems and that PR of such structures should be carefully analysed.

The mean contributions of the translational, expansive and rotational strains to the total strain of a triangle were found to be 23.2 ± 0.05%, 77.3 ± 0.05% and 0.0 ± 0.05%, respectively. The corresponding correlations between those and a triangle's total strain were 0.24 ± 0.005, 0.93 ± 0.005, and 0.03 ± 0.005, respectively. We also found a significant anti-correlation of –0.10 between the expansive strains of neighbouring triangles, as well as a considerable anti-correlation of –0.35 between their rotations. The latter is directly related to antiferromagnetic-like rotational dynamics in such systems, discussed in granular systems.^31^,^39^–^41^ We emphasise that, by imposing static determinacy, we constrained movement in our systems and, had the triangles been rigid, they would have no degrees of freedom to deploy and not displace. Thus, the displacements we observe arise from local expansions and contractions, which fits well with the correlations we observe.

We also searched for correlations between the sign of the PR and local microstructural descriptors. Such correlations, if found, could lead to informed design of auxetic disordered structures. However, we found none in this study. It is possible that such correlations have been washed out by the strong anti-correlation between nearest-neighbour expansive strains. To study this issue in more depth, a similar study should be carried out on systems of fully rigid triangles^9^ with fewer boundary constraints. We conjecture that such systems would show stronger correlations between the local PR and the translational and rotational strains. The latter is directly related to the local structural rotational disorder, defined in ref. 26 and 27 as the symmetric part of the local tensor **Q**_*t*_(*r[combining right harpoon above]*) and we therefore expect that such systems would show local structure–PR correlations.

## Conflicts of interest

There are no conflicts to declare.
